# Choosing safe and effective anticoagulation to treat idiopathic ovarian vein thrombosis: using first principles of deep vein thrombosis management to treat a rare diagnosis: a case report and review of the literature

**DOI:** 10.1186/s13256-023-03876-3

**Published:** 2023-04-21

**Authors:** Erwin Yii, Hexiang Yao, Ming Yii

**Affiliations:** 1 Department of Vascular Surgery, Eastern Health, Box Hill, VIC Australia; 2 Healthbound Family Practice, Bentleigh East, VIC Australia; 3 Department of Vascular Surgery, Monash Health, Clayton, VIC Australia

**Keywords:** Ovarian vein thrombosis, Deep vein thrombosis, Anticoagulation, Case report

## Abstract

**Background:**

Ovarian vein thrombosis (OVT) often presents in the post-partum period and is associated with significant complications including inferior vena cava extension, pulmonary embolism, sepsis, and renal obstruction. Idiopathic OVT is rare, and no consensus has been agreed upon regarding its diagnosis and management. This case presents a patient who was diagnosed with idiopathic OVT and was treated with apixaban. A literature review was performed collating reported cases of idiopathic OVT to form a recommendation regarding optimal management and follow up.

**Case presentation:**

A 42-year-old Chinese woman presenting with right lower quadrant pain underwent a CT abdomen after urinary tract obstruction was excluded on ultrasound. She was subsequently diagnosed with an idiopathic 35 mm ovarian vein thrombus (OVT) given no history of primary coagulopathy nor secondary aetiology. A literature review was performed collating 18 case reports with method of diagnosis and management summarized. Treatment alternatives included low molecular weight heparin, warfarin, rivaroxaban and apixaban. Most were diagnosed after work up for suspected renal calculus or appendicitis. Follow up imaging was performed from between 6 weeks to 6 months after initiation of anticoagulation.

**Conclusions:**

Direct oral anticoagulants were an effective treatment for OVT, however warfarin should be commenced in those suspected of antiphospholipid syndrome awaiting confirmation or exclusion of the diagnosis.

## Case report

### Background

Ovarian vein thrombosis (OVT) often presents in the post-partum period and is associated with significant complications including inferior vena cava extension, pulmonary embolism, sepsis, and renal obstruction [1]. Other secondary causes include malignancy, systemic inflammatory diseases, sepsis and primary thrombophilia [1]. However, idiopathic OVT is rare, and no consensus has been agreed upon regarding its diagnosis and management. Recommendations have been offered to manage episodes like other deep venous thromboses in the lower limbs [2]. Yet this remains unsupported by randomised trials in the setting of low incidence. This case presents a patient who was diagnosed with idiopathic OVT and was treated with apixaban. A literature review was performed collating reported cases of idiopathic OVT to form a recommendation regarding optimal management and follow up based on current experience.

### Case presentation

A 42-year-old Chinese woman presented to her general practitioner with 1 day of worsening right lower quadrant pain, described as a persistent dull ache, radiating to the right flank and groin. She was afebrile and without nausea or vomiting. She had no vaginal bleeding or discharge, nor symptoms of urinary tract infection.

She is gravida 2, para 2 and otherwise a healthy non-smoker without any significant past medical history. She does not take any regular medication. Her alcohol consumption is minimal. She has no history of thrombosis or hereditary thrombophilia. She has no gynaecological history nor previous miscarriage. She is a homemaker, living with her husband and two children and is independent of her activities of daily living. She has no significant medical family history.

On examination she demonstrated slight discomfort on deep palpation over the right groin, below McBurney’s point. She did not have rebound tenderness. Her vital signs were within normal limits with a heart rate of 65 beats per minute, blood pressure of 128/68 and temperature of 36.3 °C. Her neurological examination was unremarkable with normal power and sensation in both upper and lower limbs. Her urine dipstick was unremarkable with no nitrites or leucocytes found. Routine blood tests were unremarkable with a haemoglobin of 124 g/L, white cell count of 6.4 × 10^9^/L, thyroid stimulating hormone of 2.54 mU/L, eGFR of > 90 and INR of 1.0.

**Fig. 1:**
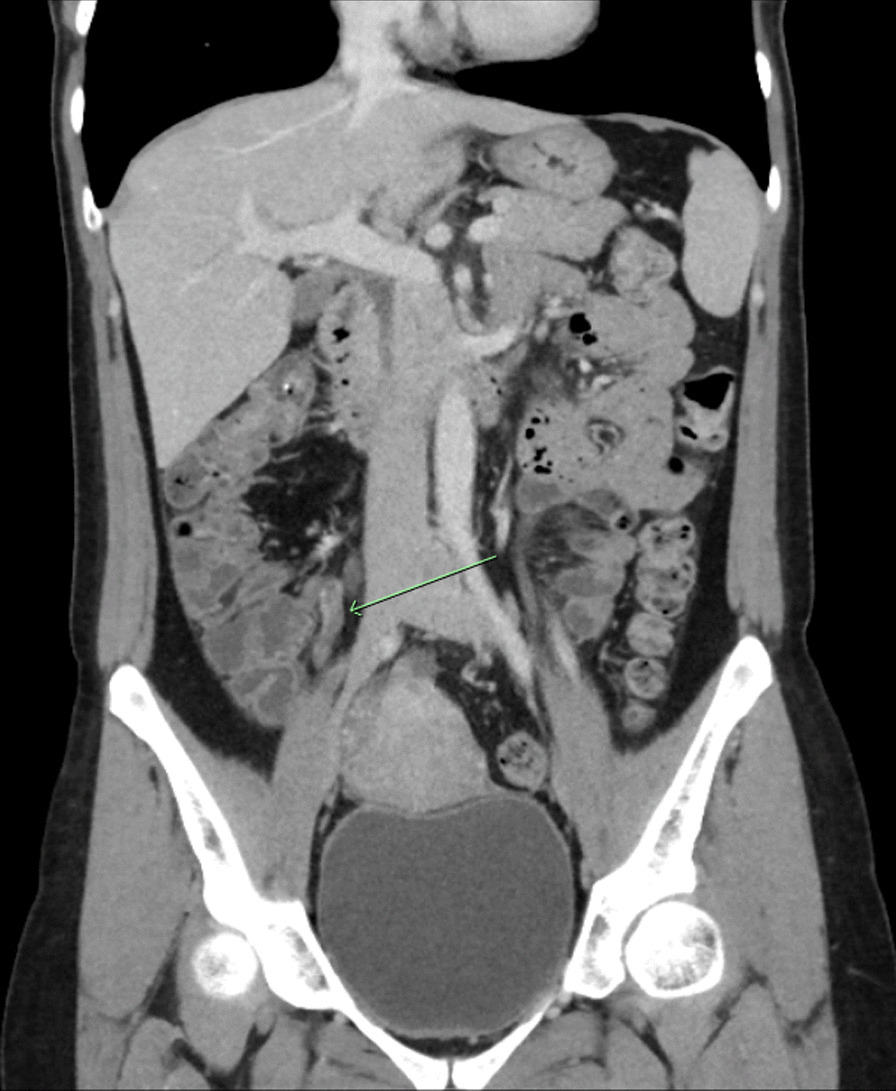
CT abdomen of ovarian vein thrombosis (green arrowing pointing to thrombus)

Her initial renal tract ultrasound ruled out urinary tract obstruction and calculi. A CT abdomen was arranged given her pain had not resolved with simple analgesia, which revealed a 35 mm segment thrombus of the lower right ovarian vein as depicted in Fig. 1.

She was commenced on apixaban 2.5 mg twice daily and referred to vascular surgery for ongoing management and monitoring. Coagulation testing for hereditary thrombophilia including prothrombin gene mutation, factor V Leiden mutation, antithrombin III deficiency, protein C, protein S and antiphospholipid antibodies were negative. Her beta-human chorionic gonadotropin pregnancy test was negative. Her groin pain subsequently improved after 3 months of anticoagulation with resolution of thrombus demonstrated on repeat Doppler ultrasound. Six months since presentation, she remains symptom free and is planned for a follow up CT angiogram to monitor progress.

## Discussion

This case describes a 42-year-old woman who presents with idiopathic OVT who was successfully treated with apixaban with resolution of her symptoms. Ovarian vein thrombosis commonly presents in the post-partum period and has been well described in this setting. This case is unique in that idiopathic OVT is rare and its description in the literature is relatively scant. As such there is no consensus on the optimal management of idiopathic OVT. This case describes successful management using apixaban demonstrating a viable treatment regime for this novel presentation.

Ovarian vein thrombosis (OVT) was first described by Austin in 1952 [3]. Complications can be life-threatening and include inferior vena cava (IVC) extension, pulmonary embolism, sepsis, and renal obstruction [1]. Classically a disease of the post-partum period, OVT more commonly presents in the right versus the left ovarian vein given compression by the uterus during pregnancy and its drainage to the IVC being at an acute angle which increases venous stasis [2]. This is consistent with Virchow’s triad of endothelial injury, hypercoagulability, and venous stasis which are all exacerbated in pregnancy. Secondary causes of OVT have been reported in settings of hypercoagulability including sepsis, pyelonephritis, malignancy, Behcet’s disease, inflammatory bowel disease and post-operatively. Even so, idiopathic episodes of OVT have been reported, though rare.

A literature review was performed using key words “ovarian vein thrombosis” on OVID Medline yielding 265 results. After screening of titles and abstracts 18 case reports were identified of idiopathic aetiology as presented in Table 1. Given limited evidence in the literature, there is no consensus on the diagnostic approach and management. As ultrasound (US) is operator dependent with variable accuracy, CT and MRI have been recommended as the optimal diagnostic imaging modality. Treatment follows the principles of deep venous thrombosis (DVT) with anticoagulation for 3 to 6 months [2].

**Table 1:** Literature review of reported cases of idiopathic ovarian vein thrombosis

Author and Year	Age	Clinical Presentation	Diagnosis	Management
Al-Shokri *et al.* 2021 [6]	32F	Chronic abdominal pain	Abdominal and pelvic gadolinium enhanced MRI	Warfarin for 6 months with resolution of symptoms
Alalqam* et al.*2019 [7]	42F	Left iliac fossa and periumbilical pain–tender on examination	Abdominal doppler, confirmed by CT	Warfarin for 6 months Doppler 1 year post showed resolution of thrombus
Basit* et al.* 2020 [8]	41F	Sharp bilateral iliac fossa pain radiating to pubic symphysis. Associated nausea and constipation	Abdominal and pelvic CT with IV and PO contrast	LMWH switched to apixaban for 6 months. Symptomatic improvement on day 3
Christy* et al.* 2021 [9]	30F	Bilateral lower pelvic pain and nausea	Abdominal and pelvic CT	Anticoagulation and repeat imaging in 40–60 days
Doherty* et al.* 2015 [10]	29F	Left lower quadrant pain for 8 months	Abdominal USS	Warfarin for 6 weeks. Doppler 2 months post showed resolution of thrombus
Garcia 2017 [11]	35F	Left flank pain for 2 days	Abdominal and pelvic CT with IV contrast	Rivaroxaban with resolution of symptoms by week 3. Repeat USS at week 6 and 12 demonstrated recanalization
Harris* et al.* 2012 [12]	53F	Right flank pain for 1 week	Abdominal and pelvic CT with IV contrast	Warfarin for 5 months with INR between 2 and 3. CT follow up at 5 months showed persistent thrombus with no extension. Warfarin subsequently discontinued
Heavrin 2008 [13]	29F	Left lower quadrant abdominal pain, nausea and vomiting for 3 days	Abdominal and pelvic CT with contrast	Anticoagulation for 6 months. No complications at 18 months follow up
Khishfe* et al.* 2016 [14]	30-39F	Colicky groin pain with associated nausea and point tenderness	Abdominal and pelvic CT (looking for nephrolithiasis)	Warfarin (Coumadin) and oral antibiotics
Kodali* et al.* 2016 [15]	40F	Right lower quadrant pain associated with nausea	Abdominal and pelvic CT (looking for appendicitis)	LMWH followed by warfarin (INR 2–3) for 6 months with symptomatic improvement by day 2
Li* et al.* 2021 [16]	33F	Acute on chronic right lower quadrant pain for 2 years	Laparotomy	Thrombus removed at surgery, commenced rivaroxaban for 3 months
Markus* et al.* 2022 [17]	27F	Left lower quadrant pain for 1 week associated with nausea	Laparoscopy followed by abdominopelvic CT and MRI	Apixaban for 3 months with symptomatic improvement after 24 h
Murphy* et al.* 2006 [18]	27F	Right lower quadrant pain radiating to right flank associated with anorexia and nausea	Abdominal and pelvic CT	Anticoagulation–unspecified
Stafford* et al.* 2010 [19]	42F	Sudden onset central abdominal and right iliac fossa pain associated with nausea	Abdominal and pelvic CT	Unfractionated heparin followed by warfarin on discharge, 2-month USS showed complete resolution
Tahir* et al.* 2021 [20]	42F	Severe sharp throbbing abdominal pain for 4 days associated with eating	Abdominal and pelvic CT	LMWH with rivaroxaban on discharge
Takazawa* et al.* 2022[21]	63F	Mild lower abdominal pain for 4 weeks	Abdominal and pelvic CT	Rivaroxaban for 8 months with improvement of symptoms after 1 month
Trang* et al.* 2020 [22]	47F	Non-specific back pain radiating to anterior abdomen associated with left lower quadrant pain	Abdominal and pelvic CT with IV contrast	LMWH with improvement in 24 h, discharged with rivaroxaban for 3 months. Follow up CTAP at 2 months showed complete resolution of thrombus
Yıldırım* et al.* 2005 [23]	36F	Nausea and abdominal pain for 2 days	Abdominal USS followed by abdominal and pelvic CT	Unfractionated heparin with warfarin with symptomatic improvement. Follow up CT on day 40 showed a persistent thrombus

*CT* computer tomography; *MRI* magnetic resonance imaging; *LMWH* low molecular weight heparin; *USS* ultrasound; *INR* international noramlised ratio; *CTAP* computer tomography abdomen pelvis; *PO* per oral; *IV* intravenous

Review of the literature identified 18 cases reporting idiopathic OVT. Patients did not have associated malignancy, surgery, sepsis, pelvic disease or primary coagulopathies. Lower abdominal pain was the most common presenting complaint, often associated with nausea. As such, most were diagnosed on abdominal and pelvic CT with differentials of appendicitis or renal calculi. Two cases were diagnosed on exploratory surgery. Management, however, was more variable. Some patients were immediately commenced on warfarin while others were bridged with unfractionated or low molecular weight heparin. Others were commenced on a direct oral anticoagulant (DOAC) such as apixaban or rivaroxaban. Duration of anticoagulation ranged from 6 weeks to 8 months. Common to each case was a prompt symptomatic response upon commencing anticoagulation with follow up occurring in 6 weeks to 6 months with accompanying CT or US imaging to demonstrate recanalization or resolution of the thrombus.

Direct oral anticoagulants are the choice of therapy for venous thromboembolism (VTE) given their superior bleeding safety profile and non-inferiority for preventing recurrent VTE compared to warfarin–recent studies also demonstrate non-inferiority of DOACs in cancer associated VTE [4]. However, DOACs are contraindicated in triple-positive antiphospholipid syndrome (APS) patients, where warfarin is the anticoagulant of choice. Because a diagnosis of APS requires positive antibodies on repeat blood tests 12 weeks apart [5], the safest anticoagulant agent to commence patients on is warfarin where there is suspicion of APS–with low molecular weight or unfractionated heparin bridging–until the diagnosis can be excluded.

This case reports a rare episode of idiopathic OVT with successful treatment on initiation of apixaban. Combining the experience of known cases of idiopathic OVT to date, recommendations have been offered based on the best evidence currently available.

## Conclusion

Idiopathic OVT is a rare condition. Given limited evidence in the literature, a range of treatments have been trialled, generally following the principles of DVT management with anticoagulation and follow up imaging which have been summarized in Table 2. While this review has demonstrated that DOACs can effectively treat idiopathic OVT, warfarin may be a more prudent choice in patients with suspected antiphospholipid syndrome until it can be confidently excluded to reduce risk of thrombus extension and embolism.

**Table 2:** Management alternatives for treating idiopathic ovarian vein thrombosis

Management	Duration	Follow up for resolution
Warfarin (INR 2–3)	6 weeks to 6 months	USS or CT at 6 weeks to 6 months
LMWH switched to apixaban	6 months	Clinical review at 3 and 6 months
LMWH switched to rivaroxaban	3 months	CTAP at 2 months
LMWH switched to warfarin (INR 2–3)	6 months	Clinical review at 6 months
UFH switched to warfarin	1 to 2 months	USS or CT at 1 to 2 months
Rivaroxaban	8 months	USS at week 6 and 12
Apixaban	3 months	Clinical review at 3 months

*CT* computer tomography; *LMWH* low molecular weight heparin; *UFH* unfractionated heparin; *INR* international noramlised ratio; *CTAP* computer tomography abdomen pelvis

## Data Availability

The datasets used and/or analysed during the current study are available from the corresponding author on reasonable request.

## References

[1] Jenayah AA, Saoudi S, Boudaya F (2015). Ovarian vein thrombosis. Pan Afr Med J.

[2] Tait C, Baglin T, Watson H (2012). British Committee for Standards in Haematology. Guidelines on the investigation and management of venous thrombosis at unusual sites. Br J Haematol.

[3] Austin OG (1956). Massive thrombophlebitis of the ovarian veins; a case report. Am J Obstet Gynecol.

[4] Raskob GE, van Es N, Verhamme P, Hokusai VTE Cancer Investigators (2018). Edoxaban for the treatment of cancer-associated venous thromboembolism. N Engl J Med.

[5] Miyakis S, Lockshin MD, Atsumi T (2006). International consensus statement on an update of the classification criteria for definite antiphospholipid syndrome (APS). J Thromb Haemost.

[6] Al-Shokri SD, Sardar S, Shajeedha Ameerudeen F, Abdul MM (2021). Non-pregnancy-related ovarian vein thrombosis: a rare cause of chronic abdominal pain. Qatar Med J.

[7] Alalqam MM, Al Abbas R, Abualsaud AS, AlQattan AS, Almabyouq F (2019). The challenges of diagnosing idiopathic ovarian vein thrombosis: case report. Int J Surg Case Rep.

[8] Basit A, Kaur P, Villanueva DM, Tahir M, Sonnenschine M (2020). Idiopathic bilateral ovarian vein thrombosis in a non-pregnant healthy patient: a case report and review of the literature. Cureus..

[9] Christy J, Jarugula D, Kesari K, Kunadi A (2021). Idiopathic bilateral ovarian vein thrombosis. BMJ Case Rep.

[10] Doherty K, New M (2015). Idiopathic ovarian vein thrombosis in a nonperipartum patient. Obstet Gynecol.

[11] Garcia R, Gasparis AP, Loh SA, Labropoulos N (2017). A rare case of idiopathic bilateral ovarian vein thrombosis. J Vasc Surg Venous Lymphat Disord.

[12] Harris K, Mehta S, Iskhakov E, Chalhoub M, Maniatis T, Forte F, Alkaied H (2012). Ovarian vein thrombosis in the nonpregnant woman: an overlooked diagnosis. Ther Adv Hematol.

[13] Heavrin BS, Wrenn K (2008). Ovarian vein thrombosis: a rare cause of abdominal pain outside the peripartum period. J Emerg Med.

[14] Khishfe BF, Sankovsky A, Nasr I (2016). Idiopathic ovarian vein thrombosis: a rare cause of abdominal pain. Am J Emerg Med.

[15] Kodali N, Veytsman I, Martyr S, Lu K (2017). Diagnosis and management of ovarian vein thrombosis in a healthy individual: a case report and a literature review. J Thromb Haemost.

[16] Li W, Cao S, Zhu R, Chen X (2021). Idiopathic ovarian vein thrombosis causing pulmonary embolism: case report and literature review. J Int Med Res.

[17] Markus J, van der Weiden RMF (2022). Laparoscopic diagnosis of idiopathic left ovarian vein thrombosis in a 27-year-old woman. JRSM Open..

[18] Murphy CS, Parsa T (2006). Idiopathic ovarian vein thrombosis: a rare cause of abdominal pain. Am J Emerg Med.

[19] Stafford M, Fleming T, Khalil A (2010). Idiopathic ovarian vein thrombosis: a rare cause of pelvic pain—case report and review of literature. Aust N Z J Obstet Gynaecol.

[20] Tahir N, Sherchan R, Farooqi A, Shrestha J, Jeelani HM (2021). Idiopathic ovarian vein thrombosis: a rare cause of abdominal pain. Cureus..

[21] Takazawa I, Matsubara D, Takahashi Y, Matsubara S (2022). Bilateral ovarian vein thrombosis without underlying conditions: a case report. J Obstet Gynaecol Res.

[22] Trang N, Kalluri M, Bajaj T, Petersen G (2020). Idiopathic left ovarian vein thrombosis. J Investig Med High Impact Case Rep..

[23] Yildirim E, Kanbay M, Ozbek O, Coskun M, Boyacioglu S (2005). Isolated idiopathic ovarian vein thrombosis: a rare case. Int Urogynecol J Pelvic Floor Dysfunct.

